# The role of transcription factor Runx2 in tumor infiltrating T cells

**DOI:** 10.1186/2051-1426-1-S1-P23

**Published:** 2013-11-07

**Authors:** Rina Mbofung, Weiyi Peng, Chengwen Liu, Chunyu Xu, Shruti Malu, Yan Yang, Wencai Ma, Zhiqiang Wang, Willem Overwijk, Eric Davis, Brendan Lee, Patrick Hwu

**Affiliations:** 1 Graduate School of Biomedical Sciences, University of Texas Health Science Center at Houston, Houston, TX, USA; 2 Melanoma Medical Oncology, University of Texas MD Anderson Cancer Center, Houston, TX, USA; 3 Lymphoma and Myeloma, University of Texas MD Anderson Cancer Center, Houston, TX, USA; 4 Molecular and Human Genetics, Baylor College of Medicine, Houston, TX, USA

## 

Adoptive T cell therapy (ACT) is a promising treatment for melanoma patients with a clinical response rate of about 50%. However, half of patients treated do not respond to this therapy, underlining the need for improvement . One of the limitations of ACT is the poor effector function of transferred T cells influenced by the immunosuppressive tumor microenvironment. In order to identify pathways which may contribute to this observation, we used a murine ACT model in which mice bearing established B16 tumors were treated with Pmel T cells which recognize the melanoma antigen gp100 in the context of H-2Db. Pmel T cells were recovered on day 6 and 13, after transfer, from the tumor and spleen of treated mice and their gene expression patterns were compared. We found that 720 genes were differentially expressed by T cells recovered from the tumor site compared to those recovered from the spleen. Amongst the differentially expressed genes were several transcription factors, including Runx2, Rora, E2F1 and Tcf7. After an initial in vivo screen, Runx2 overexpressing Pmels conferred a worse antitumor effect when compared to the control Pmels (median tumor size 30.7 vs 20.7 mm^2^ respectively on day 7 after T cell transfer, p<0.05). We also found fewer numbers of circulating Pmels in mice that received Runx2 overexpressing Pmels when compared to mice that received control Pmels. In addition, there was decreased accumulation of Runx2 overexpressing Pmels at the tumor site when compared to the control Pmel (Median luciferase output of 2.0X10^7^ vs 9.0X10^7^ photons/s/cm^2^/sr, respectively on day 6 after T cell transfer, p value =0.042). To further interrogate the role of Runx2 in T cells, we assessed the production of IFN-γ after stimulation in vitro with either plate bound anti-CD3 or gp100 expressing tumor cells. We found that IFN-γ production was comparable between Runx2 overexpressing Pmels and control Pmels after anti-CD3 stimulation. However, IFN-γ production was impaired in Runx2 overexpressing Pmels upon stimulation with tumor cells. Furthermore, in vitro characterization also revealed that Runx2 overexpressing Pmels have decreased proliferation and display an apoptotic phenotype. Taken together, our studies suggest that Runx2 regulates apoptosis, proliferation and IFN-γ production in tumor reactive T cells. Further studies to mechanistically understand these findings are ongoing.

